# Supplementary material for: Altered microRNA Transcriptome in Cultured Human Liver Cells upon Infection with Ebola Virus
Source: Int J Mol Sci. 2021 Apr 6;22(7):3792. doi: 10.3390/ijms22073792 (PMC8038836; doi:10.3390/ijms22073792)
Supplement: Supplementary file 1 [file ijms-22-03792-s001.zip › Supplementary_File/D_ KEGG_Analysis_Results/16-30nt_pathway_Mayinga-24h-Huh7_vs_Control-24h-Huh7_up.mature_mirna_targets/hsa_GeneRatioDotPlot.pdf]

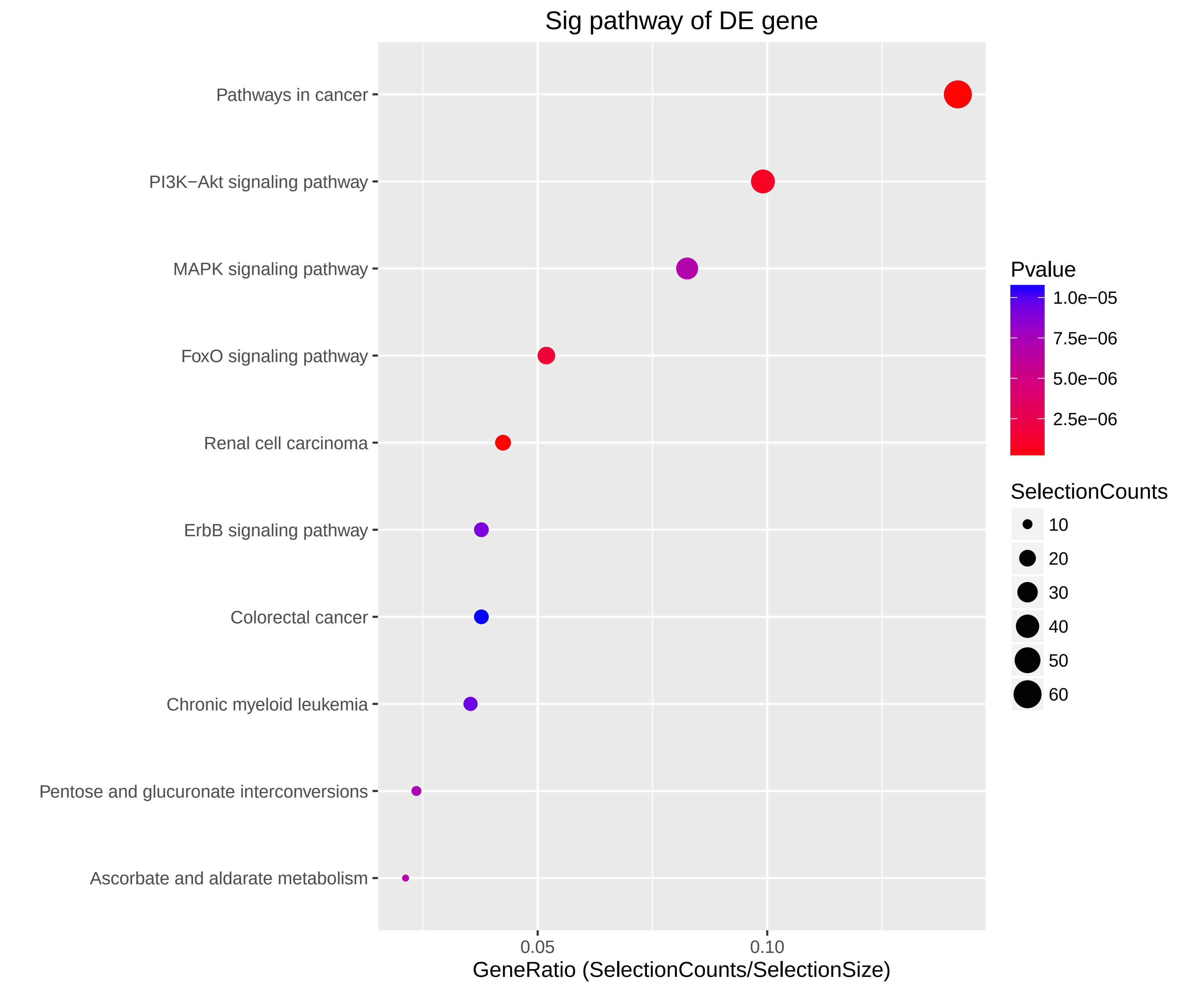

Sig pathway of DE gene
Pathways in cancer
PI3K−Akt signaling pathway
MAPK signaling pathway
FoxO signaling pathway
Renal cell carcinoma
ErbB signaling pathway
Colorectal cancer
Chronic myeloid leukemia
Pentose and glucuronate interconversions
Ascorbate and aldarate metabolism
0.05
0.10
GeneRatio (SelectionCounts/SelectionSize)
Pvalue
1.0e−05
7.5e−06
5.0e−06
2.5e−06
SelectionCounts
10
20
30
40
50
60
